# Evaluating cortical responses to speech in children: A functional near-infrared spectroscopy (fNIRS) study

**DOI:** 10.1016/j.heares.2020.108155

**Published:** 2021-03-01

**Authors:** Rachael J. Lawrence, Ian M. Wiggins, Jessica C. Hodgson, Douglas E.H. Hartley

**Affiliations:** a National Institute for Health Research (NIHR), Nottingham Biomedical Research Centre, Ropewalk House, 113 The Ropewalk, Nottingham NG1 5DU, United Kingdom; b Hearing Sciences, Division of Clinical Neuroscience, School of Medicine, University of Nottingham, Nottingham NG7 2UH, United Kingdom; c Nottingham University Hospitals NHS Trust, Derby Road, Nottingham NG7 2UH, United Kingdom; d Lincoln Medical School - Universities of Nottingham and Lincoln, Charlotte Scott Building, University of Lincoln, Lincoln LN6 7TS, United Kingdom

**Keywords:** Auditory cortex, Children, fNIRS, Functional near-infrared spectroscopy, Neuroimaging, Speech comprehension

## Abstract

Functional neuroimaging of speech processing has both research and clinical potential. This work is facilitating an ever-increasing understanding of the complex neural mechanisms involved in the processing of speech. Neural correlates of speech understanding also have potential clinical value, especially for infants and children, in whom behavioural assessments can be unreliable. Such measures would not only benefit normally hearing children experiencing speech and language delay, but also hearing impaired children with and without hearing devices. In the current study, we examined cortical correlates of speech intelligibility in normally hearing paediatric listeners. Cortical responses were measured using functional near-infrared spectroscopy (fNIRS), a non-invasive neuroimaging technique that is fully compatible with hearing devices, including cochlear implants. In nineteen normally hearing children (aged 6 – 13 years) we measured activity in temporal and frontal cortex bilaterally whilst participants listened to both clear- and noise-vocoded sentences targeting four levels of speech intelligibility. Cortical activation in superior temporal and inferior frontal cortex was generally stronger in the left hemisphere than in the right. Activation in left superior temporal cortex grew monotonically with increasing speech intelligibility. In the same region, we identified a trend towards greater activation on correctly vs. incorrectly perceived trials, suggesting a possible sensitivity to speech intelligibility *per se*, beyond sensitivity to changing acoustic properties across stimulation conditions. Outside superior temporal cortex, we identified other regions in which fNIRS responses varied with speech intelligibility. For example, channels overlying posterior middle temporal regions in the right hemisphere exhibited relative deactivation during sentence processing (compared to a silent baseline condition), with the amplitude of that deactivation being greater in more difficult listening conditions. This finding may represent sensitivity to components of the default mode network in lateral temporal regions, and hence effortful listening in normally hearing paediatric listeners. Our results indicate that fNIRS has the potential to provide an objective marker of speech intelligibility in normally hearing children. Should these results be found to apply to individuals experiencing language delay or to those listening through a hearing device, such as a cochlear implant, fNIRS may form the basis of a clinically useful measure of speech understanding.

## Highlights

• fNIRS has the potential to provide an objective measure of speech understanding.
• Left superior temporal activation grows monotonically with speech intelligibility.
• fNIRS responses also vary with speech intelligibility in other cortical regions.
• Right posterior middle temporal regions exhibit relative deactivation.
• The amplitude of deactivation is greater in more difficult listening conditions.

## 1 Introduction

Proficient speech and language skills promote the development of effective social functioning and academic achievement (Mlcakova et al., 2012). Whilst the processing of speech and language at the cortical level is complex, functional neuroimaging techniques are facilitating an ever-increasing understanding of the neural mechanisms involved (Hickok and Poeppel 2000; Scott and Johnsrude, 2003). Furthermore, imaging of cortical language areas may provide an objective way of assessing speech understanding in specific cohorts such as infants and children, in whom behavioural assessments alone may be unreliable (Anderson et al., 2017).

Functional near-infrared spectroscopy (fNIRS) is an increasingly popular, non-invasive optical technique that can be used safely and repeatedly for studying cortical function (Saliba et al., 2016). It has many advantages over other neuroimaging techniques, which include being: 1) acoustically silent, which is of specific importance when analysing responses to auditory stimuli; 2) portable, allowing for subjects to be imaged in both clinical and research environments; 3) relatively resistant to head movements so that infants and children can be scanned whilst awake and sitting on a parent's knee; 4) compatible with hearing devices, including cochlear implants (CIs). Indeed, in normally hearing individuals and deaf CI users alike, fNIRS has reliably measured cortical responses to speech in both adult (Anderson et al., 2017b; McKay et al., 2016; Olds et al., 2016; Wiggins et al., 2016) and paediatric populations (Blasi et al., 2014; Sevy et al., 2010). fNIRS images the haemodynamic response to neuronal activity in the brain via the use of near-infrared light (Boas et al., 2014). Low-power near-infrared light is directed through the scalp and into the cortex; the intensity of the light returning to the surface of the scalp is then detected. Changes in the concentration of oxygenated haemoglobin (HbO) and deoxygenated haemoglobin (HbR) can be measured, which are then subsequently interpreted as an indirect reflection of neuronal activity.

Evidence from our group (Lawrence et al., 2018) and that of others (Pollonini et al., 2014; Olds et al., 2016) has demonstrated that fNIRS can measure cortical correlates of speech intelligibility in adults. Specifically, consistent with fMRI studies (Binder et al., 2004; Davis and Jonsrude, 2003; Zekveld et al., 2006), a positive association was found between speech intelligibility and cortical activation of superior temporal regions (Lawrence et al., 2018; Pollonini et al., 2014; Olds et al., 2016). Although we observed significant activation in superior temporal cortex bilaterally and did not directly test for inter-hemispheric differences, our group found that these intelligibility-sensitive regions were more spatially extensive in the left hemisphere than in the right (Lawrence et al., 2018). However, for an undetermined reason, right-hemisphere responses appeared better able to account for individual differences in speech intelligibility. Beyond superior temporal cortex, we also identified regions in which activation varied non-linearly with intelligibility. For example, in left inferior frontal gyrus (LIFG), activation peaked in response to heavily degraded, yet still somewhat intelligible, speech. This pattern of results is consistent with a role for the LIFG in supporting speech comprehension under effortful listening conditions (Wild et al., 2012; Wijayasiri et al., 2017).

Evidence from a range of neuroimaging methods suggests that the left hemisphere plays the major role in processing of speech and language (Homae, 2014; Knecht et al., 2000; Corballis et al., 2009; Pujol et al., 1999; Szaflarski et al., 2012). Left-lateralised fNIRS responses to speech have been observed in infancy, and even in neonates (Peña et al., 2003; Dehaene-Lambertz et al., 2002). Interestingly, fNIRS responses to clear and intelligible speech in infants have been shown to lateralise to the left hemisphere, whereas responses to unintelligible speech (i.e. the same speech played backwards) activate both cerebral hemispheres equally (Peña et al., 2003; Sato et al., 2012). In older children (aged 6 - 12 years), left-sided channels targeting posterior temporal regions have shown significantly greater fNIRS activation to normal speech compared with right-sided corresponding channels (Mushtaq et al., 2019).

It has been suggested that it may be disadvantageous to have linguistic functions evenly distributed across the hemispheres (Bishop, 2013), with any inefficient processing possibly leading to reduced performance on language tasks and, subsequently, affecting language development. However, it remains to be established whether a more left-lateralised processing network improves language ability and furthermore whether different lateralisation profiles occur at different stages of maturity. Research specifically aimed at examining language lateralisation patterns in children between infancy and adulthood is currently lacking, and is indeed required in order to assess cortical mechanisms involved in speech and language processes across the full spectrum of development.

Clinically, measurement of cortical activation to speech could also prove useful to help identify infants and children who have delayed speech and language skills, including those who exhibit speech delay, either with normal hearing, or following hearing loss. Early identification of these children at risk of developing speech and language delay can help clinicians to intervene early to maximize the benefit of any rehabilitation approach during a sensitive period of their development.

Here we expand on our previous work in adults to assess cortical correlates of speech intelligibility in a group of normally hearing school-aged children. We concentrate on this age group as the vast majority of studies to date have been conducted on either adults or infants, with a paucity of data collected in between. We principally target superior temporal and inferior frontal brain regions to establish whether i) activation in superior temporal regions correlates with speech intelligibility, ii) activation is consistently left-lateralised in school-aged children listening to speech at different levels of intelligibility, and iii) the LIFG exhibits an elevated response to degraded, compared to clear, speech. In order to study these cortical correlates of speech understanding, we presented normally hearing 6 - 13 year-old children with both clear and noise-vocoded speech (Shannon et al., 1995) that was parametrically varied from intelligible to unintelligible.

## 2 Materials and methods

### 2.1 Participants and ethical approval

Nineteen healthy child volunteers (mean age 9.4 years, range 6–13 years, 13 females, 6 males) were recruited to the study. The design was approved by the University of Nottingham Faculty of Medicine and Health Sciences Research Ethics Committee. Written informed consent was obtained from the accompanying parents or guardians of all participants. Participants had no known psycho-motor impairments or specific language differences (e.g. dyslexia), and all had self-reported/parent-reported normal hearing. Intelligence was assessed using the Wechsler Abbreviated Scale of Intelligence (WASI-II) (Weschler, 2011), with the group mean age-corrected intelligence quotient (IQ) ranked at the 88th percentile (range 55th to 99.9th percentile). All participants were native English speakers with normal or corrected-to-normal vision. Most participants were right handed (17 out of 19) as assessed using a shortened version of the Edinburgh Handedness Inventory (Oldfield, 1971).

### 2.2 Equipment

Testing was conducted in a sound-attenuated room with the lighting dimmed. Participants were seated approximately 75 cm from a visual display unit. A Genelec 8030A loudspeaker mounted immediately above and behind the display was used to present the auditory stimuli, at a level of 65 dB SPL (A-weighted root-mean-square level averaged over the duration of each sentence, measured at the listening position using a Brüel & Kjær Type 2250 sound level metre with the participant absent). Brain activity was non-invasively measured using a Hitachi (Tokyo, Japan) ETG-4000 continuous-wave fNIRS system. The ETG-4000 measures simultaneously at wavelengths of 695 nm and 830 nm (sampling rate 10 Hz) and uses frequency modulation to minimize crosstalk between channels and wavelengths (Scholkmann et al., 2014). A dense sound-absorbing screen was placed between the fNIRS equipment and the listening position, resulting in a steady ambient noise level of 38 dB SPL (A-weighted). During the main fNIRS task, participants entered their responses using an “RTbox” button box (Li et al., 2010). The experiment was implemented in MATLAB (MathWorks, Natick, MA) using the Psychtoolbox-3 extensions (Brainard, 1997; Kleiner et al., 2007; Pelli, 1997).

### 2.3 Speech stimuli

Speech materials consisted of recordings of Bamford-Kowal-Bench (BKB) sentences (Bench et al., 1979) spoken by a male talker. We used eight-channel noise vocoding to create sentences that were acoustically degraded. Noise vocoding reduces spectral clarity while preserving temporal envelope cues (Shannon et al., 1995). The eight channels were spaced approximately equally along the basilar membrane (Greenwood, 1990) and spanned an overall bandwidth of 180–8000 Hz. Channel filtering was performed using 6th-order digital elliptic filters applied consecutively in the forward and reverse directions to avoid phase distortion (MATLAB *filtfilt* function). Within-channel amplitude envelopes were extracted by half-wave rectification followed by zero-phase low-pass filtering at 160 Hz using a 1st-order elliptic filter (applied consecutively in the forward and reverse directions). Following subsequent manipulation (see below), each envelope was then applied to a white-noise carrier and bandpass filtered using the same filters as used for analysis. Input and output root-mean-square (RMS) levels were matched on a within-channel basis, before summation across channels to arrive at the final stimulus.

To parametrically vary the intelligibility of the vocoded stimuli, we manipulated the depth of envelope modulation within each vocoder channel by raising the extracted envelopes to a fractional power (same exponent for all channels). In previous work with adult participants, we established that using envelope exponents of 0.000, 0.149, 0.212, 0.297 and 1.000 was suitable to target group-mean intelligibility levels of 0, 25, 50, 75 and 100% keywords correct, respectively (Lawrence et al., 2018). In the present study, we retained the stimulus conditions with envelope exponents of 0.000, 0.212 and 1.000 (notionally corresponding to 0, 50 and 100% intelligibility in adults), and throughout this paper we refer to these as Noise, Noise Vocoded Low (NVLow), and Noise Vocoded High (NVHigh), respectively. An envelope exponent of 0.000 (Noise) resulted in a steady speech-shaped noise containing no linguistic information, while an exponent of 1.000 (NVHigh) left the original speech envelope unaltered (i.e. comparable to a standard eight-channel vocoder). In retaining these envelope exponents, we were mindful that the school-aged participants in the present study would not necessarily achieve the same level of speech understanding as we observed previously in adults (Lawrence et al., 2018). We therefore included an additional reference condition of clear (i.e. non-vocoded) speech, which was expected to be fully intelligible. On these trials, the vocoder was bypassed, but the speech signal was passed through the same filter-bank to ensure equivalent bandwidth across all stimulation conditions.

### 2.4 fNIRS task procedure

An event-related design was used based on previous studies in our laboratory (Lawrence et al., 2018; Wijayasiri et al., 2017). We simultaneously performed fNIRS imaging along with a behavourial task. Each event in the experiment corresponded to the presentation of a single sentence. Participants were presented with 18 BKB sentences per degraded stimulus condition, 9 sentences in the clear speech condition, and 18 silent baseline trials. The reason for including fewer trials in the clear speech condition was because we anticipated that fNIRS responses would be inherently more robust in this condition (compared to the degraded speech conditions) and we wanted to keep the overall testing duration manageable for our younger participants. All trial types were presented in randomised order. The stimulus-onset asynchrony (SOA; the time between the onset of auditory stimulation on one trial and the next) was randomly varied in the range 6–12 s (average SOA: 9 s; average offset-to-onset gap: 7.4 s). It should be noted that, especially for SOAs towards the lower end of this range, a degree of temporal overlap was to be expected between the prolonged haemodynamic response to one trial and the next. However, by randomising the SOA, and on the assumption of linearity in the summation of the haemodynamic response to individual events, it becomes possible to accurately and efficiently estimate the response to each trial type, despite this temporal overlap (Dale, 1999). Building on the established use of rapid event-related designs in the fMRI literature, the validity of rapid event-related fNIRS was demonstrated by Plichta et al. (2007), and such designs have subsequently been used widely across diverse domains of fNIRS research. The imaging lasted approximately 18 min in total.

During imaging, participants were instructed to look at a fixation cross presented on a uniform background, while sitting as still as possible to minimize motion artefacts. Instead of participants repeating the sentences verbally, we used a task that required a simple button press response in order to minimize any artefacts due to overt speech production. In this task, 0.5 s after each stimulus ended a probe word appeared on the display. The probe word was, with equal probability, either a word that had featured in the presented BKB sentence or a replacement foil word, chosen to rhyme with one of the true keywords. Foil words were equally likely to fall towards the start, middle, or end of the sentence. Participants were required to indicate by a button press whether the probe word had appeared in the sentence just heard. “Yes” and “No” labels were presented towards either side of the display accordingly. The orientation of these labels was fixed throughout an individual's session; however, the side that corresponded to a “Yes” response was alternated for even- and odd-numbered participants. Participants had up to 2 s to respond; otherwise a missed response was recorded. On silent trials, in lieu of the speech-based task, participants were simply instructed to either “Press yes” or “Press no” at random. To ensure that participants were confident with the procedure, a short familiarisation session was conducted before the optode array was placed on the participant's head. A secondary purpose of this familiarisation session was to provide participants with listening experience of degraded (noise-vocoded) speech.

### 2.5 fNIRS measurements

fNIRS measurements were made with a total of 20 optodes arranged in two 2 × 5 arrays (each containing 5 sources and 5 detectors). The arrays were placed on both sides of the participant's head. We aimed to primarily measure cortical activation bilaterally in temporal and frontal cortex. To ensure consistency of optode placements across individuals, we followed a fixed protocol. The international 10–20 system (Jasper, 1958) was used to guide and standardise optode placement: the central optical source in the bottom row of the array was positioned in vertical alignment with the preauricular point, with the central detector optode in the row above aligned towards position Cz. The inter-optode spacing was fixed at 30 mm. Once the position of the optode array was completed, a photograph was taken of the final placement for reference purposes. During testing, participants were instructed to remain still and keep head movements to a minimum to reduce motion artefacts in the recorded data.

Optode positions were not digitised at the time of data collection due to unreliable behaviour of the Polhemus 3D digitizer within the confines of the sound booth in which testing took place. For the purposes of results visualisation, optode positions were subsequently digitised on a separate group of 12 child volunteers of comparable age, wearing the same array placed on the head following the same protocol. Indicative visual overlay of results on the cortical surface is based on the mean optode positions across the 12 volunteers.

### 2.6 Analysis of fNIRS data

Analysis of the fNIRS data was performed in MATLAB (MathWorks, Natick, MA) using functions provided in the HOMER2 package (Huppert et al., 2009) together with custom scripts. The analysis was performed in a similar manner to previous studies conducted in our laboratory (Anderson et al., 2017b; Lawrence et al., 2018; Mushtaq et al., 2019; Wiggins et al., 2016; Wiggins and Hartley, 2015; Wijayasiri et al., 2017). In brief, the following steps were performed:

**Exclusion of channels with poor signal quality:** we used the scalp coupling index (SCI) to identify and exclude channels suffering from poor optode – scalp contact (Pollonini et al., 2014). We excluded channels with SCI <0.06, chosen to exclude only the worst 5% of channels in our dataset. We note that this SCI threshold is much lower than the value of 0.75 taken by Pollonini et al. to indicate satisfactory optode – scalp contact. However, Pollonini et al. used a higher-density optode array, providing a degree of spatial overlap amongst the fNIRS measurement channels. Such an overlap was not possible with the sparse optode array used in the present study, meaning that we needed to be cautious to avoid excluding large swathes of the measurement array. In previous studies using the same setup, we have found excluding only the worst 5% of channels to be a useful rule-of-thumb, striking an appropriate balance between the wish to include only high-quality channels in the analysis versus the risk of committing type II (false negative) statistical errors if the effective sample size is too heavily reduced due to extensive channel exclusions.

**Conversion to optical density**: the measured light intensity levels were converted to optical density using the HOMER2 *hmrIntensity2OD* function, a standard step in fNIRS data analysis (Huppert et al., 2009).

**Motion-artefact correction:** after the raw fNIRS intensity signals had been converted, wavelet filtering was conducted using the HOMER2 *hmrMotionCorrectWavelet* function, an implementation of a technique described by Molavi and Dumont (2012). In this approach, the influence of motion artefacts is reduced by eliminating outlying wavelength coefficients, which are assumed to represent artefacts. We excluded wavelet coefficients lying further than 0.719 times the interquartile range below the first or above the third quartiles. If the wavelet coefficients are assumed to be normally distributed, this equates to the α = 0.1 threshold used in fNIRS motion artefact correction method evaluations (Brigadoi et al., 2014).

**Bandpass filtering:** the optical density signals were band-pass filtered between 0.02 and 0.5 Hz to attenuate low-frequency drift and cardiac oscillations.

**Conversion to estimated changes in haemoglobin concentrations:** optical density signals were then converted to estimated changes in the concentration of HbO and HbR through application of the modified Beer-Lambert Law (Huppert et al., 2009). A default value of 6 was used for the differential path-length factor at both wavelengths. Note that the continuous-wave fNIRS system used in the present study allows for the estimation only of relative changes in haemoglobin concentrations across conditions and not absolute concentrations.

**Isolation of the functional haemodynamic response:** we applied the haemodynamic modality separation (HMS) algorithm described by Yamada et al. (2012) to isolate the functional component of the haemodynamic signal and suppress systemic physiological interference. This algorithm attempts to separate functional and systemic signals based on the assumption that the correlation between HbO and HbR will be different in each case. Although this approach does not accurately account for all statistical properties of the noise typically found in fNIRS data (Huppert, 2016), in previous studies we have found application of this algorithm to be beneficial to the detection of auditory cortical activation (Lawrence et al., 2018; Wiggins et al., 2016; Wijayasiri et al., 2017); in particular, application of the HMS algorithm was shown to substantially improve the test-retest reliability of auditory fNIRS measurements (Wiggins et al., 2016). Note that HbO and HbR become statistically redundant after application of the HMS algorithm; subsequent analysis and visualisation of results is based on HbO for convenience.

**Quantification of response amplitude:** to quantify fNIRS response amplitude we used a general linear model (GLM) approach previously described in Wijayasiri et al. (2017) and Lawrence et al. (2018). The GLM was applied to the continuous data collected over the duration of the imaging session. The design matrix included a set of three regressors (corresponding to the canonical haemodynamic response plus its first two temporal derivatives) for each experimental condition, plus a further set for the silent trials. The purpose of including the temporal derivative terms is to allow flexibility in the model to capture small differences in the delay and/or duration of the observed haemodynamic response (as compared to the canonical response). Each trial was modelled as a short epoch corresponding to the actual period of auditory stimulation on that trial (mean duration 1.64 s; audio muted on silent trials). Within each condition, the canonical and temporal-derivative regressors were serially orthogonalised with respect to one another (Calhoun et al., 2004). Model estimation was performed using the two-stage ordinary least squares procedure described by (Plichta et al., 2007), which incorporates a correction for serial correlation (Cochrane and Orcutt, 1949). While inclusion of the temporal-derivative terms allows the model to capture small differences in the delay and/or duration of the haemodynamic response (as might occur across brain regions or experimental conditions), any such systematic differences can induce bias in the estimation of response amplitude if the amplitude is estimated from the canonical term alone (Calhoun et al., 2004). We therefore used the ‘derivative-boost’ technique (Calhoun et al., 2004; Steffener et al., 2010), which combines the canonical (nonderivative) and the derivative terms of the model to derive an estimate of response amplitude that is unbiased by differences in the delay and/or duration of the observed haemodynamic response between conditions.

### 2.7 Statistical analyses

#### 2.7.1 Behavioural data during fNIRS task

Repeated-measures analyses of variance (RM-ANOVAs) were carried out using IBM SPSS Statistics for Windows Version 25.0 software (IBM Corp., Armonk, New York). The Greenhouse-Geisser correction for non-sphericity was applied where necessary. The behavioural results were analysed using a one-way RM-ANOVA with within-subject factor “stimulation condition” (four levels: Noise, NVLow, NVHigh and Clear). Separate analyses were performed for accuracy and response time. Polynomial contrasts were used to identify trends in the data across stimulation conditions. Note that, in keeping with Binder et al. (2004), response time data from all trials were included in the analysis, regardless of whether the participant's response was correct or incorrect. This is because the time taken to arrive at a decision could potentially have a bearing on the observed brain activity, regardless of whether a correct response was ultimately achieved. In practice, almost identical results were obtained if the calculation of mean response time was instead confined to correct responses only.

#### 2.7.2 fNIRS data

Our primary statistical analysis aimed to establish the relationship between intelligibility of the speech stimulus and fNIRS response amplitude on a channel-wise basis. Therefore, in the first instance the estimated response amplitude for each experimental condition was contrasted against the silent baseline condition. A linear mixed model (LMM) was then used to analyse the contrast values for each channel (West et al., 2006). Since in the present study we did not have available estimates of speech intelligibility at an individual-participant level, we treated the four experimental conditions (Noise, NVLow, NVHigh, Clear) as a series of ordered categorical predictors going from lower to higher intelligibility.

To enable us to detect possible non-linear relationships between brain activation and speech intelligibility, we included the polynomial expansion of stimulus condition up to 2nd order. Serial orthogonalisation was applied to the polynomial terms such that the 0th order (constant), 1st order (linear) and 2nd order (quadratic) fixed effects were independent of one another and could be separately and straightforwardly interpreted (Büchel et al., 1998). The effect of the serial orthogonalisation was such that maximal variance was at each stage assigned to the lower-order term (e.g. the constant term), with subsequent terms (e.g. the linear and quadratic terms) tested on the remaining variance. Each LMM additionally included a random intercept effect to model between-subject variability.

We performed secondary group-level analyses to obtain further insight into the nature of the fNIRS-measured cortical activation. We tested for an effect of the veracity of trial-by-trial perception on fNIRS response amplitude by re-running the original GLM analysis, but now including additional regressors to separately estimate response amplitude on “correct” and “incorrect” trials. We then used a LMM to test for main effects of perceptual veracity and stimulus condition, as well as a possible veracity x stimulus condition interaction. The LMM included only those conditions in which correct speech understanding was possible, but not guaranteed, namely the NVLow and NVHigh conditions.

An additional aim was to assess whether speech-evoked fNIRS responses in school-aged children show evidence of being preferentially lateralised to one hemisphere over the other. A laterality index has been used in studies with other neuroimaging modalities, most prominently fMRI, to indicate hemispheric dominance and this is calculated using the following formula: (Q_LH_-Q_RH_)/(Q_LH_+Q_RH_) where Q_LH_ and Q_RH_ are representative measures acquired from the left and right hemispheres (Seghier et al., 2008). Any calculated value usually ranges between −1 (indicating pure right hemispheric dominance) and +1 (for pure left hemispheric dominance). However, a drawback of this formula is that it remains valid only if all measures are positive in value, which for our data was not always the case. Therefore, following the approach taken by Mushtaq et al. (2019), we assessed laterality using a simplified approach in which fNIRS response amplitude was contrasted between symmetrically matching channels from the left and right hemispheres. Again, we used a LMM to test for main effects of hemisphere and stimulus condition, as well as for a possible hemisphere x stimulus condition interaction. Data from all four stimulus conditions were included in this analysis.

In all fNIRS analyses, we accounted for multiple comparisons by applying the false discovery rate (FDR) method (Benjamini and Hochberg, 1995) across channels. We used the original formulation of the FDR procedure, which assumes independence or slight positive dependency across tests, as recommended for fNIRS data analysis by Singh and Dan (2006). Statistical significance was assessed against an FDR-corrected threshold of q < 0.05.

## 3 Results

### 3.1 Behavioural results

**Fig. 1:**
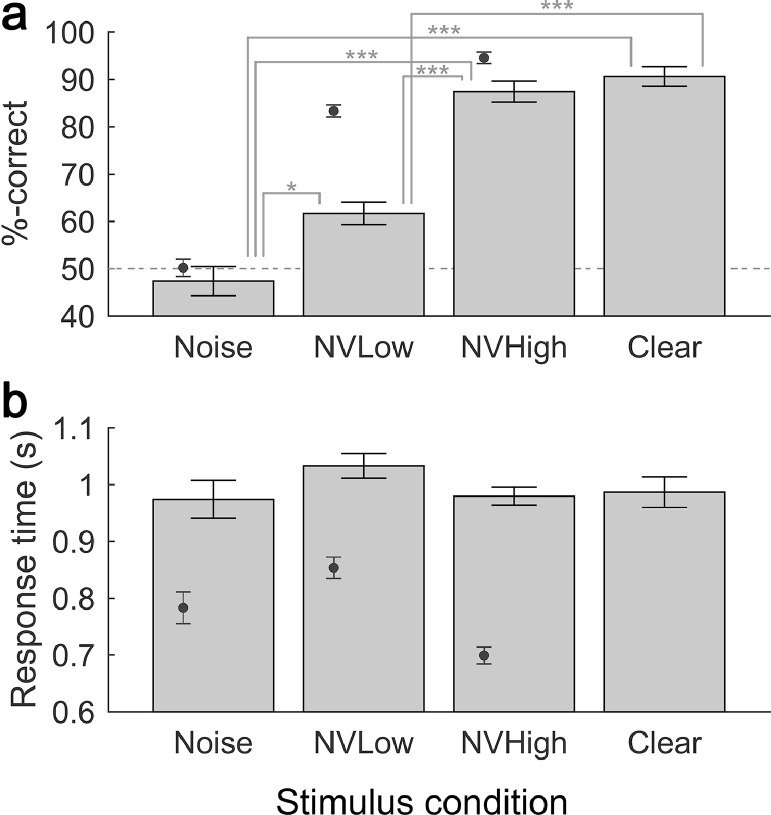
**Behavioural results collected concurrently with fNIRS imaging.** Mean accuracy (a) and response time (b) is shown for each of the four stimulation conditions. The bars show results for children in the present study, while the inset markers show comparative data for normally hearing adults acquired in our previous study (Lawrence et al., 2018). Error bars show ±1 standard error of the mean (corrected for repeated measures). The dashed horizontal line in (a) represents chance performance. Asterisks indicate significant pairwise differences in post-hoc tests following a significant RM-ANOVA omnibus test result (* *p* < .05, ** *p* < .01, *** *p* < .001, Bonferroni-corrected).

Accuracy and response time data for the behavioural task (conducted simultaneously with fNIRS imaging) are plotted in Fig. 1. The bars represent the behavioural results for *n* = 19 children in the current study, while the inset markers correspond to comparative data for *n* = 24 normally hearing adults taken from a previous study (Lawrence et al., 2018). Missed responses were rare, occurring on less than 3% of trials overall.

As expected, and in concordance with our established adult data (Lawrence et al., 2018), accuracy in distinguishing true keywords from rhyming foil words increased monotonically as the intelligibility of the speech stimulus increased. Since accuracy was close to 100% in some conditions, a rationalised arcsine transform was applied prior to statistical analysis (Studebaker, 1985). Accuracy was significantly above chance (50%) in all conditions except for the Noise condition (one-sample *t*-tests, *p* < .05 after Bonferroni correction). A RM-ANOVA confirmed a significant effect of stimulation condition (*F*(3, 54) = 51.836, *p* <. 001) with polynomial trend analysis revealing significant linear and cubic components (*p* <. 01). Post-hoc tests revealed that all stimulation conditions differed significantly from one another (*p* < .05 in all cases, Bonferroni-corrected), with the exception of the NVHigh and Clear conditions. Although not subjected to formal statistical analysis, it is apparent that the adult participants in the study of Lawrence et al. (2018) displayed markedly superior performance when listening to noise-vocoded speech (NVLow and NVHigh conditions) compared to the child participants in the present study.

In contrast to the accuracy scores, mean response time did not differ significantly across stimulation conditions (*F*(2.164, 38.955) = 0.846, *p* > .05; Fig. 1b). Again, although not subjected to formal statistical analysis, we observe that when compared to the adult data from Lawrence et al. (2018), children demonstrate markedly longer response times across all stimulus conditions that were common across both studies.

### 3.2 fNIRS results

#### 3.2.1 Channel-wise analysis of relationships with speech intelligibility

**Fig. 2:**
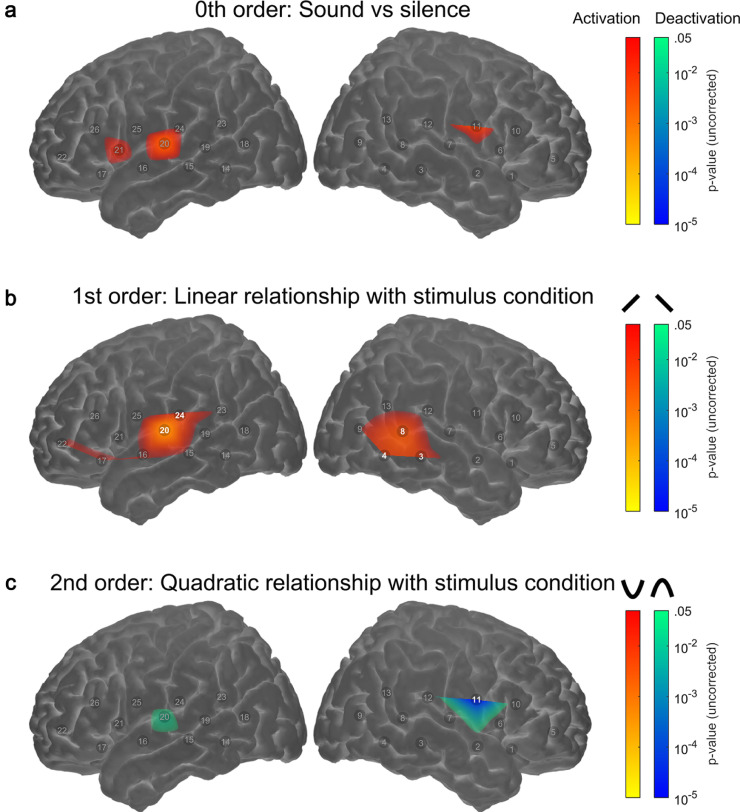
**Channel-wise relationship between fNIRS response amplitude and speech stimulus intelligibility**. Rows a to c show the results of statistical significance testing (uncorrected *p*-values, thresholded at *p* < .05) for 0th-order, 1st-order (linear) and 2nd-order (quadratic) effects, respectively. Individual channels exhibiting significant effects after correction for multiple comparisons (*q* < 0.05, FDR corrected) are highlighted. Note the maps are interpolated from single-channel results and the overlay on the cortical surface is for illustrative purposes only.

The results of the primary analysis testing for systematic relationships between fNIRS response amplitude and stimulus intelligibility are shown in Fig. 2. A corresponding table of statistical results is provided as supplementary information (Supplementary Table 1). Fig. 2a shows the 0th- order effect, i.e. overall activation or deactivation in response to sound vs. silence, irrespective of stimulus intelligibility. Although there was some evidence of cortical activation in channels overlying left superior temporal cortex (Ch#20), and left (Ch#21) and right (Ch#11) frontal areas, this activation failed to reach statistical significance after correction for multiple comparisons.

Fig. 2b identifies channels that exhibited a significant linear relationship with speech intelligibility (1st-order effect). Such channels were found to lie principally over left superior temporal cortex (Ch#20,24), as well as in a cluster of posterior temporal channels in the right hemisphere (Ch#3,4,8). No linear relationship between speech intelligibility and fNIRS response amplitude was observed in right superior temporal cortex.

Fig. 2c identifies channels in which activation depended non-linearly on speech intelligibility (2nd-order/quadratic effect). The only channel to exhibit a significant quadratic effect (after correction for multiple comparisons) was Ch#11, a channel in the right frontal lobe identified as overlying the rolandic operculum. This channel exhibited an “inverted-U” response profile (maximal activation at intermediate speech intelligibility). The rolandic operculum has previously been reported to play a role in sensori-motor control during phonological processing (Heim and Friederici, 2003; Vigneau et al., 2006).

#### 3.2.2 Response profiles in regions-of-interest

**Fig. 3:**
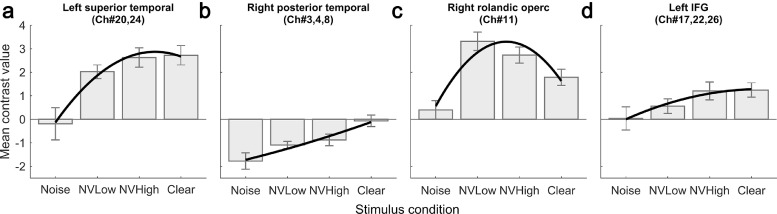
**Mean contrast values (i.e. estimated response amplitude relative to silence; arbitrary units) in select regions of interest.** Error bars show ±1 standard error of the mean (corrected for repeated measures). Bold lines indicate the overall LMM fit for each region including polynomial expansion of stimulus condition up to 2nd (quadratic) order.

In order to detail how fNIRS response amplitude varied with speech stimulus intelligibility in different parts of the brain, Fig. 3 plots mean fNIRS contrast values (relative to silence) against stimulus condition for select regions-of-interest (ROIs). The first three ROIs were defined in a post-hoc manner by selecting clusters of adjacent channels that exhibited significant linear or quadratic relationships with stimulus condition (cf. Fig. 2b and c) and which all showed a qualitatively similar response profile within a given ROI. The resulting ROIs are descriptively labelled left superior temporal (Ch#20,24), right posterior temporal (Ch#3,4,8), and right rolandic operculum (Ch#11). In addition, we included a fourth ROI overlying the LIFG (Ch#17,22,26). In a previous study with adults (Lawrence et al., 2018), we found that activation in the LIFG peaked in response to heavily degraded, yet still potentially intelligible, speech, consistent with the notion that increased LIFG activity could be a marker for effortful listening (Wild et al., 2012; Wijayasiri et al., 2017). We wanted to investigate whether the same might be true for the paediatric participants in the present study.

In the left superior temporal ROI, considered to principally overlie the auditory cortex, activation increased monotonically as the intelligibility of the speech stimulus improved, with the most pronounced step occurring between the Noise and NVLow conditions (Fig. 3a). In the right posterior temporal ROI, the general trend was of relative deactivation compared to the silent baseline condition (Fig. 3b), with the strength of the deactivation linearly increasing as the intelligibility of the speech signal decreased i.e. from the Clear to the Noise condition. In the right rolandic operculum, positive activation (relative to silence) was seen in all conditions (Fig. 3c), with the strength of that activation increasing monotonically as speech intelligibility was reduced from the Clear to the NVLow condition. However, activation in the right rolandic operculum then fell off steeply in the Noise condition, in which the stimulus was stripped of all meaningful linguistic information. In the LIFG, rather than the quadratic “inverted-U” response profile that we had observed previously in adults (Lawrence et al., 2018), in the present paediatric study responses in this region grew monotonically with increasing speech intelligibility, being greatest in the most intelligible stimulus conditions (NVHigh and Clear).

**Fig. 4:**
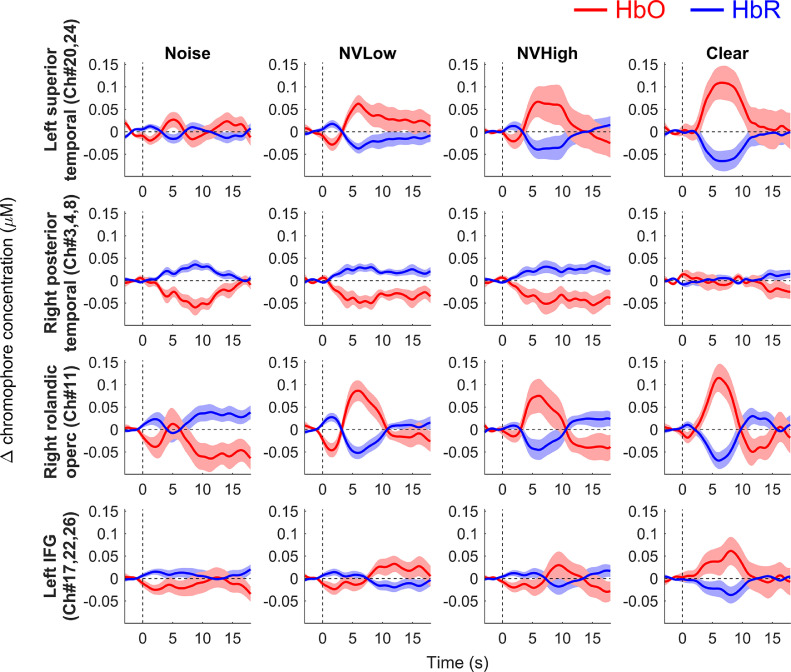
**Grand average event-related haemodynamic time courses.** The red and blue traces show estimated changes in the concentration of HbO and HbR, respectively. Shading indicates ±1 standard error of the mean across participants. Note that, prior to averaging across participants, the mean response to silent trials was subtracted out to derive overlap-reduced event-related responses. (For interpretation of the references to colour in this figure legend, the reader is referred to the web version of this article.)

Fig. 4 plots grand-average event-related haemodynamic time courses across the group of participants for reference. To estimate the shape of the underlying haemodynamic response, while reducing the influence of temporal overlap in the response across consecutive trials, the mean fNIRS response to silent trials was subtracted out (on a per-participant basis) to derive overlap-reduced event-related responses for each speech condition. The observed responses are broadly consistent with the results of our primary statistical analysis (as shown in Fig. 3 and described above), e.g. left superior temporal responses that increase in amplitude when moving from less intelligible to more intelligible stimulus conditions, and apparent “deactivation” (negative going HbO traces) in the right posterior temporal region. However, in some cases, convergence between the event-related responses plotted in Fig. 4 and the statistical response profiles plotted in Fig. 3 is less clear, e.g. in the right rolandic operculum, where the statistically robust “inverted-U” response profile is not clearly identifiable in the event-related responses. The precise reasons for this discrepancy are unclear, but may reflect procedural differences between the two types of analyses (e.g. in Fig. 4, rather than subtracting a model-derived estimate of the amplitude of the response on silent trials, the full silent-trial response waveform has been subtracted from the response to each speech condition on a moment-by-moment basis).

#### 3.2.3 Relationship between fNIRS response amplitude and perceptual veracity

**Fig. 5:**
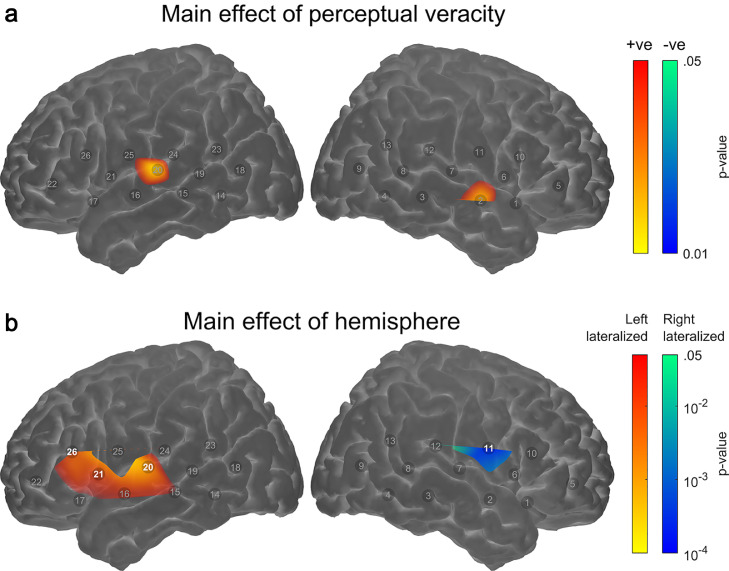
**Secondary group-level fNIRS analyses**. (a) Main effect of perceptual veracity (i.e. the contrast between correct vs. incorrect trials) for the NVLow and NVHigh conditions. (b) Main effect of hemisphere (assessment of laterality across all stimulation conditions). In each case, the colourmap shows uncorrected *p*-values, thresholded at *p* < .05. Individual channels showing a significant effect after correction for multiple comparisons (*q* < 0.05, FDR corrected) are highlighted. Note that the maps are interpolated from single-channel results and the overlay on the cortical surface is for illustrative purposes only.

Fig. 5a shows the results of channel-wise significance testing for a main effect of trial-by-trial perceptual veracity, i.e. the contrast between correctly versus incorrectly perceived trials (restricted to the NVLow and NVHigh conditions as previously described). A corresponding table of statistical results is provided as supplementary information (Supplementary Table 2). Five participants had to be excluded from this analysis because they did not give any incorrect responses in one or other of the NVLow and NVHigh conditions, preventing estimation of the associated fNIRS response amplitude. Although not statistically significant (after multiple-comparisons correction), there was a trend towards greater activation in left (Ch#20) and right (Ch#2) superior temporal regions, suggesting that activation in these regions may be stronger when a speech signal is perceived correctly. In this analysis, no channels showed a statistically significant main effect of stimulus condition, nor a statistically significant interaction between perceptual veracity and stimulus condition (not plotted).

#### 3.2.4 Hemispheric lateralisation assessment

We tested for a difference in fNIRS response amplitude between corresponding channels in the left and right hemispheres to assess lateralisation of cortical activity. Fig. 5b shows the results of channel-wise significance testing for a main effect of hemisphere. The tests were two-tailed, allowing for the detection of both left- and right-lateralised activation (plotted separately for clarity of visual presentation). A corresponding table of statistical results is provided as supplementary information (Supplementary Table 2). We observed a statistically significant left-hemispheric dominance for activation in superior temporal and inferior frontal regions (Ch#20,21,26), whereas activation in channels overlying the rolandic operculum lateralised to the right hemisphere (Ch#11). All of the aforementioned lateralisation effects were significant (*q* < 0.05) after multiple-comparisons correction. In this analysis (in which responses were analysed in pairs of corresponding channels from the left and right hemispheres), no channel-pairs showed a statistically significant main effect of stimulus condition, nor a statistically significant interaction between hemisphere and stimulus condition (not plotted).

## 4 Discussion

### 4.1 Cortical correlates of speech intelligibility and hemispheric lateralisation of language processing

With the use of noise vocoding, we varied speech intelligibility whilst measuring cortical activation with fNIRS in a cohort of normally hearing children. Compared with previous adult and paediatric fNIRS and fMRI studies, we report both confirmatory and novel findings. Specifically, in terms of behavioural speech perception, as expected, children showed poorer processing efficiency compared with adults, revealed by consistently lower task accuracy and longer response times (Fig. 1). It has been suggested that poor processing efficiency amongst children primarily represents attentional and cognitive immaturity (Hartley and Moore, 2002; Hartley et al., 2003; Hill et al., 2004).

We confirm that activation in superior temporal regions (i.e. “auditory” cortex) correlates with speech intelligibility (Binder et al., 2004; Davis and Jonsrude, 2003; Lawrence et al., 2018; Pollonini et al., 2014; Olds et al., 2016; Zekveld et al., 2006). Furthermore, we observed a trend towards greater temporal-lobe activation on correctly perceived trials (Fig. 5a), suggesting a possible sensitivity to speech intelligibility *per se*, rather than simply the changing acoustic properties across stimulus conditions. However, unlike in our previous study in adults (Lawrence et al., 2018), the contrast between correctly and incorrectly perceived trials did not survive correction for multiple comparisons across channels in the present study. While this could reflect a genuine difference between children and adults, alternatively it might simply reflect a slightly smaller effective sample size, and therefore lower statistical power, in the present study.

One difference between the current findings and those of our previous study in adults (Lawrence et al., 2018) is that here we observed a significant linear relationship between speech intelligibility and cortical activation in left superior temporal cortex only (Fig. 2b), as opposed to in bilateral superior temporal cortex in adults. In the present study, we also found statistically significant lateralisation of responses towards the left hemisphere in superior temporal and inferior frontal regions in school-age children. This finding of left-hemispheric lateralisation of responses to speech has been observed in numerous infant (Bortfeld et al., 2007, 2009; Peña et al., 2003; Sato et al., 2012) and paediatric (Mushtaq et al., 2019) fNIRS studies, in addition to both adult (Frost et al., 1999) and paediatric (Weiss-Croft and Baldeweg, 2015) fMRI studies.

The observed left-hemispheric dominance for language processing in paediatric, but not adult, fNIRS studies may relate to the brain-scalp (B-S) distance. In children, significantly greater B-S distances have been observed in frontal and temporal regions in comparison with other cortical regions, a finding particularly apparent for the right hemisphere (Beauchamp et al., 2011). Larger B-S distances are associated with an increased cerebrospinal fluid (CSF) volume in the inner table of the cranium (Beauchamp et al., 2011) and CSF is known to attenuate the fNIRS signal by reducing spatial resolution (Custo et al., 2006). Left-sided temporal activation in response to speech stimuli may therefore appear more robust than for right temporal cortex with paediatric fNIRS imaging due to the potentially shorter B-S distance in the left hemisphere (Beauchamp et al., 2011). The B-S distance has also been shown to increase with age (Beauchamp et al., 2011). In terms of the lack of lateralisation of language in adult fNIRS studies (Lawrence et al., 2018), this may be explained by the finding of a negative correlation between increasing B-S distance and the concordance between fMRI and fNIRS measurements (Cui et al., 2011). Hence, the findings of Beauchamp et al. (2011) offer a possible explanation for the difference in lateralisation of language observed in child and adult fNIRS studies.

In the present paediatric study we observed a statistically significant quadratic relationship between fNIRS activation and stimulus condition in cortical areas overlying the right rolandic operculum (Fig. 2c) within the frontal lobe. Established evidence suggests that rolandic areas of the brain (situated around the central sulcus, also known as the centrotemporal area) are involved in language, and more specifically, phonological processing (Heim and Friederici, 2003; Vigneau et al., 2006). Furthermore, abnormal activation in this area of the brain is associated with language impairment (Besseling et al., 2013; McGinnity et al., 2017). Rolandic epilepsy is an idiopathic form of childhood epilepsy characterised by centrotemporal spikes originating from the rolandic cortex (Besseling et al., 2013). MRI imaging studies in children affected by the condition have identified reduced functional (Besseling et al., 2013; McGinnity et al., 2017) and structural connectivity (Besseling et al., 2013) within rolandic regions, and furthermore the presence of some of these aberrant connections has been shown to correlate with reduced language performance (Besseling et al., 2013). Although we observed a significant relationship between activation in the right rolandic operculum and speech intelligibility, this is a novel finding not previously reported in the literature, hence drawing definite conclusions on fNIRS activation patterns in the rolandic operculum is difficult at present. Further fNIRS and fMRI imaging studies would be required to further investigate the significance of this finding in terms of speech and language processing.

### 4.2 Cortical correlates of effortful listening

Convergent evidence from previous studies indicates a role for the LIFG in supporting effortful listening (Alain et al., 2018). This prefrontal area has been specifically implicated in speech comprehension under difficult conditions due to demonstrating increased activation for degraded yet somewhat intelligible speech, compared to either clear speech or unintelligible noise (Davis and Johnsrude, 2003; Wild et al., 2012). Listening to noise-vocoded speech is reported to be a cognitively demanding task (Pals et al., 2013; Winn et al., 2015). In our previous adult fNIRS study, in which we parametrically varied speech intelligibility with the use of vocoded speech (Lawrence et al., 2018), we found a cluster of channels overlying the LIFG that showed a consistent trend towards a quadratic relationship with intelligibility. Activation in this cortical area was greatest at intermediate levels of intelligibility, and lowest when either the listening task was relatively easy or when the stimulus was stripped of all linguistic information. When we examined the LIFG ROI of children in this current study, we failed to observe this quadratic relationship between fNIRS activation and speech stimulus intelligibility. We instead found activation in the LIFG to increase monotonically as intelligibility of the stimulus increased (Fig. 3d). However, behavioural performance during the fNIRS task failed to reach 100 per cent correct in these children, including in the Clear condition, suggesting that perhaps even processing of clear speech retains some degree of effort for children, compared with adults. Alternatively, leftward lateralisation of activation in the IFG with age (Berl et al., 2014) has been previously shown using fMRI, suggesting the LIFG ROI in our current paediatric cohort may be yet to reach adult-like maturation and function.

In this study we also observed a significant relationship between fNIRS response amplitude and speech intelligibility in a cluster of channels overlying right posterior temporal regions (Fig. 2b). These cortical regions exhibited deactivation (relative to the silent baseline condition), with strength of deactivation being greatest for Noise and the more difficult listening conditions (Fig. 3b). In our adult study (Lawrence et al., 2018), we also observed a tendency towards deactivation under effortful listening conditions in posterior temporal regions (albeit bilateral and with a quadratic relationship with intelligibility rather than linear), and suggested that this finding likely reflected sensitivity of adult measurements to the default mode network (DMN). The DMN is a network of interconnected brain regions, including inferior parietal and lateral temporal cortex, that is preferentially more active during “rest” (Andrews-Hanna et al., 2014). Activation within the DMN is thought to reflect periods of engagement in self-generated thought (Buckner et al., 2008). Findings from this current paediatric fNIRS study align with fMRI data that has shown the DMN to deactivate during the performance of an auditory cognitive task, with the amplitude of deactivation correlating with cognitive demand and task difficulty (Mckiernan et al., 2003). The different pattern of fNIRS deactivation observed for posterior temporal regions of children (linearly increasing strength of deactivation with reducing speech intelligibility) and adults (strongest deactivation at intermediate levels of intelligibility) may be explained by children having remained more attentive to the Noise condition than adults. Due to potential cognitive immaturity (Hartley and Moore, 2002; Hartley et al., 2003; Hill et al., 2004), children may be less efficient than adults in recognising that a pure-noise stimulus lacks linguistic information. As a consequence, dedication of substantial cognitive resources to the Noise condition in children may account for the observed deactivation of the DMN for this stimulus condition, a finding not seen in our adult data (Lawrence et al., 2018). Although other studies examining paediatric cortical responses to varying speech intelligibility are lacking for comparison, established evidence supports an improvement in selective attention to an auditory stimulus with age, with older children and adults better able to ignore noise in a tone-in-noise detection task (Jones et al., 2015). The reason for a unilateral right-sided deactivation of the DMN in children in comparison to a bilateral deactivation in adults is unclear. However, longitudinal paediatric MRI data has demonstrated an increased and more distributed deactivation of the DMN (in response to an auditory comprehension task) with increasing age (Horowitz-Kraus et al., 2017). Findings from this study therefore demonstrate that fNIRS imaging may have sensitivity to components of the DMN in lateral temporal regions, and that the strength of deactivation in corresponding channels may provide a marker of the attentional demands of a challenging listening task.

### 4.3 Limitations and potential clinical impact

Our findings contribute to a body of evidence suggesting that fNIRS has the potential to objectively measure speech intelligibility (Lawrence et al., 2018; Olds et al., 2016; Pollonini et al., 2014), lateralisation of auditory responses (Gallagher et al., 2012; Arun et al., 2018; Bortfeld et al., 2007, 2009; Peña et al., 2003; Sato et al., 2012) and listening effort (Lawrence et al., 2018; Wijayasiri et al., 2017) at the cortical level. However, we specifically extend these findings to a group of individuals outside of both infancy and adulthood in terms of age.

A limitation of the present study is that, while suitable for revealing how fNIRS cortical responses in children depend on speech stimulus intelligibility in general terms, the design does not allow for the establishment of a precise predictive relationship. There are several reasons for this. Firstly, due to constraints on testing duration, we were able to include only four stimulus conditions spanning the entire range from completely unintelligible to fully intelligible speech. Secondly, although our behavioural data confirm that speech recognition accuracy increased monotonically across the four stimulation conditions, we do not have available open-set speech intelligibility scores for our child participants, and so in our statistical models we treated the four stimulation conditions as a series of ordered categorical predictors, rather than using measured intelligibility scores as a direct predictor of cortical activation. Thirdly, in the absence of open-set intelligibility scores, we conducted only group-level analyses in the present study and made no attempt to model individual differences in speech intelligibility. The establishment of a precise predictive relationship between speech intelligibility and fNIRS cortical activation in children, sensitive to individual differences, remains a subject for future study.

The ability to objectively analyse how speech and language information is received and processed by the brain would be invaluable, especially in infants and children where behavioural assessments of speech understanding can be unreliable. The early identification of speech processing patterns that deviate from “normal” has the potential to detect children who are not developing along a typical language trajectory, and furthermore, is crucial to maximise the benefit of any targeted speech and language rehabilitation package (Teagle, 2016). fNIRS shows promise as a clinical tool for assessing cortical responses in both normally hearing children and children affected by hearing loss, with or without a hearing device. With respect to cochlear implantation, if fNIRS can be utilised to inform us whether or not the speech and language processing areas of the brain are appropriately activated, then we have the opportunity to optimise the CI programming process to give individuals the best chance of developing appropriate speech and language skills. An additional limitation of this study is that the observed relationships between fNIRS cortical activation and speech intelligibility cannot be directly applied to the auditory deprived brain. Furthermore, our results along with the findings of other fNIRS studies, are yet to be applied to or interpreted at an individual level. Advances in fNIRS testing protocols and data analysis are therefore required in order to efficiently report results and their significance at the individual level, and furthermore allow for this to occur real-time in the clinical setting. Other CI compatible imaging techniques such as EEG offer improved temporal resolution and potentially reduced cost when compared with fNIRS, yet have drawbacks such as being more sensitive to head movements, which may be particularly problematic in children. Simultaneous fNIRS and EEG imaging offers analysis with high temporal resolution of both the neuronal and hemodynamic aspects of cortical activation. Combined fNIRS and EEG imaging have successfully been used in combination to not only examine, but also increase the classification accuracy of visual and auditory stimulus processing (Putze et al., 2014; Chen et al., 2015).

Despite the aforementioned limitations, this study adds to a growing body of evidence supporting the potential of fNIRS imaging in hearing research and in the future management of hearing-related conditions, either as a stand-alone modality, or in combination with other imaging techniques such as EEG.

## Declaration of Competing Interest

None.

## Appendix Supplementary materials

Image, application 1
