# Supplementary material for: Evaluating cortical responses to speech in children: A functional near-infrared spectroscopy (fNIRS) study
Source: Hear Res. 2021 Mar 1;401:108155. doi: 10.1016/j.heares.2020.108155 (PMC7937787; doi:10.1016/j.heares.2020.108155)
Supplement: Supplementary file 1 [file mmc1.docx]

**Supplementary Table 1** Statistical results for the primary fNIRS analysis (corresponding to Fig. 2 in the manuscript). Where a *p*-value is in bold, this indicates that the corresponding effect was significant after correction for multiple comparisons (*q* < .05, FDR corrected).

| Channel # | Effective sample size* | 0^th^-order (constant) effect | | 1^st^-order (linear) effect | | 2^nd^-order (quadratic) effect | |
| --- | --- | --- | --- | --- | --- | --- | --- |
|  |  | Critical *p*-value† = .0019 | | Critical *p*-value† = .0095 | | Critical *p*-value† = .0019 | |
|  |  | *t*-value | *p*-value | *t*-value | *p*-value | *t*-value | *p*-value |
| 1 | 16 | -0.683 | 0.497022 | -0.466 | 0.642821 | -0.169 | 0.866337 |
| 2 | 16 | -0.875 | 0.385267 | 0.934 | 0.353983 | 0.935 | 0.353522 |
| 3 | 15 | -1.753 | 0.085048 | 2.686 | **0.009463** | -0.095 | 0.924460 |
| 4 | 16 | -1.621 | 0.110140 | 2.764 | **0.007545** | 0.239 | 0.811900 |
| 5 | 19 | 1.511 | 0.134997 | -0.118 | 0.906325 | -0.785 | 0.434994 |
| 6 | 18 | -0.462 | 0.645313 | 1.165 | 0.248067 | -0.945 | 0.348155 |
| 7 | 17 | -0.359 | 0.720970 | -0.247 | 0.805745 | -1.126 | 0.264137 |
| 8 | 15 | -1.620 | 0.110803 | 3.037 | **0.003603** | -0.032 | 0.974378 |
| 9 | 15 | -0.252 | 0.802038 | 2.142 | 0.036471 | 1.257 | 0.213886 |
| 10 | 19 | 0.294 | 0.769775 | -0.440 | 0.660892 | -1.526 | 0.131426 |
| 11 | 19 | 2.903 | 0.004887 | 1.881 | 0.063999 | -4.539 | **0.000022** |
| 12 | 19 | 1.096 | 0.276709 | 1.600 | 0.113890 | -0.989 | 0.325969 |
| 13 | 17 | -1.608 | 0.112587 | 1.707 | 0.092531 | -0.676 | 0.501174 |
| 14 | 18 | -0.164 | 0.869999 | 1.774 | 0.080516 | 0.438 | 0.663045 |
| 15 | 19 | -0.529 | 0.598442 | 1.874 | 0.064875 | 1.070 | 0.288023 |
| 16 | 18 | 1.249 | 0.215762 | 2.057 | 0.043504 | 1.077 | 0.285038 |
| 17 | 18 | 1.402 | 0.165474 | 2.224 | 0.029437 | 0.048 | 0.961860 |
| 18 | 13 | -1.753 | 0.085856 | 0.446 | 0.657521 | -0.211 | 0.833814 |
| 19 | 16 | -0.186 | 0.852884 | 1.436 | 0.155979 | -0.420 | 0.675963 |
| 20 | 19 | 3.165 | 0.002260 | 3.686 | **0.000435** | -2.596 | 0.011401 |
| 21 | 18 | 2.451 | 0.016782 | 1.349 | 0.181673 | 0.539 | 0.591698 |
| 22 | 18 | 0.924 | 0.358500 | 2.231 | 0.028952 | -0.390 | 0.697390 |
| 23 | 17 | -0.843 | 0.402325 | 1.802 | 0.076160 | 1.480 | 0.143820 |
| 24 | 18 | 1.962 | 0.053788 | 2.796 | **0.006703** | -0.649 | 0.518209 |
| 25 | 19 | 0.876 | 0.383978 | 1.336 | 0.185656 | -0.483 | 0.630431 |
| 26 | 18 | 1.621 | 0.109596 | 0.946 | 0.347289 | -0.974 | 0.333433 |

*   After bad-channel exclusions
† Following false-discovery-rate correction across channels

**Supplementary Table 2** Statistical results for the secondary group-level fNIRS analyses (corresponding to Fig. 5a–b in the manuscript). Where a *p*-value is in bold, this indicates that the corresponding effect was significant after correction for multiple comparisons (*q* < .05, FDR corrected).

| Main effect of perceptual veracity  (cf. Fig. 5a) | | | | Main effect of hemisphere (cf. Fig. 5b) | | | |
| --- | --- | --- | --- | --- | --- | --- | --- |
| Critical *p*-value† = .0019 | | | | Critical *p*-value† = .0096 | | | |
| Channel # | Effective sample size* | *t*-value | *p*-value | Channel pair (LH – RH) | Effective sample size* | *t*-value | *p*-value |
| 1 | 11 | -0.524 | 0.603434 | 14 – 4 | 16 | 1.165 | 0.246846 |
| 2 | 11 | 2.501 | 0.016583 | 15 – 3 | 16 | 1.976 | 0.050648 |
| 3 | 9 | 0.226 | 0.822539 | 16 – 2 | 15 | 2.304 | 0.023440 |
| 4 | 10 | 0.733 | 0.468523 | 17 – 1 | 16 | 1.618 | 0.108618 |
| 5 | 13 | 1.200 | 0.236041 | 18 – 9 | 19 | -1.532 | 0.128703 |
| 6 | 12 | 1.399 | 0.168829 | 19 – 8 | 18 | 0.925 | 0.357113 |
| 7 | 11 | 0.834 | 0.409481 | 20 – 7 | 17 | 3.304 | **0.001279** |
| 8 | 9 | 1.052 | 0.300502 | 21 – 6 | 15 | 2.637 | **0.009551** |
| 9 | 9 | -0.075 | 0.940549 | 22 – 5 | 15 | 0.094 | 0.925194 |
| 10 | 13 | -0.398 | 0.692274 | 23 – 13 | 19 | 1.243 | 0.216503 |
| 11 | 13 | -0.264 | 0.792730 | 24 – 12 | 19 | 0.557 | 0.578448 |
| 12 | 13 | 1.165 | 0.249612 | 25 – 11 | 19 | -3.774 | **0.000246** |
| 13 | 11 | -1.072 | 0.289979 | 26 – 10 | 17 | 2.731 | **0.007329** |
| 14 | 12 | 0.808 | 0.423219 |  |  |  |  |
| 15 | 13 | 0.623 | 0.535931 |  |  |  |  |
| 16 | 12 | 1.309 | 0.197242 |  |  |  |  |
| 17 | 12 | -0.682 | 0.498578 |  |  |  |  |
| 18 | 9 | 1.234 | 0.226290 |  |  |  |  |
| 19 | 10 | 0.819 | 0.418176 |  |  |  |  |
| 20 | 13 | 2.563 | 0.013564 |  |  |  |  |
| 21 | 12 | -0.599 | 0.551916 |  |  |  |  |
| 22 | 12 | 1.535 | 0.132007 |  |  |  |  |
| 23 | 11 | 0.454 | 0.651976 |  |  |  |  |
| 24 | 12 | 1.322 | 0.192996 |  |  |  |  |
| 25 | 13 | 1.719 | 0.092058 |  |  |  |  |
| 26 | 12 | 0.899 | 0.373551 |  |  |  |  |

*   After bad-channel exclusions and any other participant exclusions
† Following false-discovery-rate correction across channels
